# PDCD6 is an independent predictor of progression free survival in epithelial ovarian cancer

**DOI:** 10.1186/1479-5876-10-31

**Published:** 2012-02-27

**Authors:** Dan Su, Haiyan Xu, Jianguo Feng, Yun Gao, Linhui Gu, Lisha Ying, Dionyssios Katsaros, Herbert Yu, Shenhua Xu, Ming Qi

**Affiliations:** 1 Center for Genetic and Genomic Medicine, School of Medicine, Zhejiang University, Hangzhou, Zhejiang, China; 2 Cancer Research Institute, Zhejiang Cancer Hospital, Hangzhou, Zhejiang, China; 3 Department of Obstetrics and Gynecology, Gynecologic Oncology and Breast Cancer Unit, University of Turin, Turin, Italy; 4 Departments of Epidemiology and Public Health, Yale Cancer Center, Yale University School of Medicine, New Haven, CT, USA; 5 Department of Pathology and Laboratory Medicine, University of Rochester, Rochester, NY, USA

## Abstract

**Background:**

Programmed cell death 6 (PDCD6) beside its known proapoptotic functions may be a player in survival pathways in cancer. The purpose of this study is to further explore the roles of PDCD6 in epithelial ovarian cancer.

**Methods:**

Lentiviral vector with shRNA for PDCD6 was used to investigate the effects of PDCD6 knockdown on cell growth, cell cycle, apoptosis and motility in ovarian cancer cells. Two hundred twelve epithelial ovarian cancer tissues were analyzed for mRNA expression of *PDCD6 *using RT-PCR. Associations of its expression with clinical pathological factors, progression free and overall survival were evaluated.

**Results:**

PDCD6 is highly expressed in metastatic ovarian cancer cells and positively regulates cell migration and invasion. Significantly, the level of *PDCD6 *expression in epithelial ovarian cancer correlates with clinical progression. Patients with medium or high levels of *PDCD6 *mRNA were at higher risk for disease progression, compared to those with low levels (HR, 1.29; *P *= 0.024 for medium levels; and HR, 1.57; *P *= 0.045 for high levels) after adjusting for age, disease stage, tumor grade, histologic type and residual tumor size. Kaplan-Meier survival analysis demonstrated similar results. However, no association was found between *PDCD6 *expression and overall survival.

**Conclusions:**

PDCD6 seems to play an important role in ovarian cancer progression and it may be an independent predictor of progression free survival in epithelial ovarian cancer. Further studies are needed to more completely elucidate the molecular mechanisms of PDCD6 involve in ovarian cancer progression.

## Background

Ovarian cancer is a common gynecologic malignancy and a major cause of cancer death among women in the United States [1]. The 5-year survival rate for patients with ovarian cancer is only 35% [2,3]. The high mortality of ovarian cancer is related to our inability to detect the disease early and to treat it effectively. Most of ovarian cancer patients ultimately die from tumor recurrence and metastasis, despite the fact that they initially respond to the treatment of cytoreductive surgery followed by paclitaxel and platinum-based chemotherapy. So, understanding the molecular mechanisms involving the initiation, progression, and metastasis of ovarian cancer is important for the prevention, detection, and treatment of ovarian cancer.

In our previous study, two epithelia ovarian cancer cell lines with low and high metastatic potentials were set up successively, named as HO-8910, and HO-8910 PM respectively [4,5]. Seventeen different significantly expressed proteins between them were detected by comparative proteomic analysis, of which programmed cell death 6 (PDCD6), also named as apoptosis-linked gene 2 (ALG-2), was identified [6].

PDCD6, a calcium-binding protein belonging to the penta-EF-hand protein family, participates in T cell receptor-, Fas-, and glucocorticoid-induced programmed cell death [7,8]. However, apoptosis was not blocked in mice deficient for PDCD6, suggesting PDCD6 is functionally redundant [9]. Furthermore, PDCD6 was found up-regulated in lung cancer and hepatoma tissue compared with normal tissue, also suggesting that PDCD6 beside its known proapoptotic functions may be a player in survival pathways [10]. Similarly, our previous results showed PDCD6 was over-expressed in HO-8910 PM which has higher metastatic potential, as compared with HO-8910 [6]. To further explore the function of PDCD6 in ovarian cancer, in this study, we investigated the effects of PDCD6 knockdown by short hairpin RNA (shRNA) on cell growth, cell cycle, apoptosis and motility of ovarian cancer cells. In addition, we analyzed the association between PDCD6 expression, clinical pathological factors and outcome in ovarian cancer patients.

## Materials and methods

### Cell culture

HO-8910 and HO-8910 PM cells were established by Zhejiang Cancer Hospital [4,5]. The cells were cultured in RPMI-1640 medium containing 10% newborn bovine serum, supplemented with 100 U/ml penicillin and 125 μg/ml streptomycin, and were incubated at 37°C with 5% CO_2_.

### Western blot analysis

The cells were harvested with 0.02% EDTA and 0.025% trypsin, rinsed three times in phosphate-buffered saline (PBS), and pelleted in 1.5 ml microcentrifugetubes by centrifugation. Whole-cell extracts were prepared with the Nuclear Extract Kit (Active Motif, Carlsbad, California, USA). The protein content of the cell lysate was determined by using the Bradford calorimetric assay method (Bio-Rad, Richmond, California, USA). Fifty μg of cell lysates were directly resolved by 12% SDS-PAGE and were transferred to PVDF membranes (Millipore, MA, USA). Proteins transferred to the membrane were detected by GAPDH and PDCD6 (Abcam Inc, MA, USA) primary antibodies and horseradish peroxidase-linked secondary antibodies (anti-rabbit IgG), and the proteins were then visualized by enhanced chemiluminescence reagents. BioRad Laboratories Quantity One software (BioRad, CA, USA) was used to quantify the blots, GAPDH as a loading control.

### Quantitative real-time polymerase chain reaction (qRT-PCR)

RNeasy Mini Kits (QIAGEN Inc., Valencia, CA) were used to extract total RNA from cells and tissue. RNA (5 ng) was reverse transcribed to cDNA using a SuperScript First-Strand Synthesis System for RT-PCR (Invitrogen Corp., Carlsbad, CA). Quantitative real-time PCR was performed to determine the expression level of *PDCD6 *mRNA in each tumor sample, using *GAPDH *as an endogenous control for calibration. The primer sequences were designed online (http://www.idtdna.com) and ordered from Invitrogen Corp. (Shanghai, China). The primer sequences were: TGA CCA GTT CCA CGA CAT CCT CAT (PDCD6 forward), TTG GCT CTT TCC ATG TTG TGC TGC (PDCD6 reverse), GAA GGT GAA GGT CGG AGT C (GAPDH forward), and GAA GAT GGT GAT GGG ATT TC (GAPDH reverse). Quantitative real time PCR was carried out using an ABI 7500 real-time PCR system (Applied Biosystems Inc., Foster City, CA, USA). The PCR reaction solution (25 μl) contained 5 ng/μl cDNA, 12.5 μl Power SYBR Green PCR Master Mix (Bioer Technology Inc., Hangzhou, China) and a pair of primers at a final concentration of 5 μM for *PDCD6 *and *GAPDH*. Dissociation curve analysis was performed after PCR to confirm the size of PCR products. All tumor samples were analyzed in duplicate along with negative controls. Real-time PCR results were recorded as Ct values (threshold cycle). To adjust for the total amount of cDNA used for analysis in each sample, a ΔCt was calculated based on the difference in Ct values between the target gene *PDCD6 *and the housekeeping gene *GAPDH*. ΔCt was further converted to an expression index (EI) based on the formula 2^(-ΔCt)^. Inhibition rate of *PDCD6 *was calculated by formula [1-2^(-ΔΔCt)^]*100%.

### Transfection of lentiviral vectors with shRNA for *PDCD6*

The pGCSIL-GFP-shRNA-PDCD6 lentiviral vectors (pGCSIL, a lentiviral vector) were purchased from Shanghai GeneChem Company and the target shRNA sequences as follows: 5'- cagagggtcgataaagaca-3'. pGCSIL-GFP-lentiviral vector was used as a control. The lentiviral vectors and pHelper were co-transfected into 293 T cells. The culture supernatants were collected, concentrated, and used as a virus stock. HO-8910 PM at 40%-50% confluence was inflected with lentivirus expression shRNA to the human *PDCD6 *gene or vector control. The shRNA targeting sequences was validated for the most efficient interference of PDCD6 by qRT-PCR and Immunoblotting.

### Cell proliferation assay

To assess cell proliferation effected by PDCD6, HO-8910 PM cells was infected with shRNA-PDCD6 for 72 h. Then the cells were plated at 5 × 10^3 ^cells/well in 96-well microtiter plates and cultured at 37°C with 5% CO_2_. Each 24 h, the cell viability was measured by MTT (3-[4,5-dimethylthiazol-2-yl]-2,5 diphenyl tetrazolium bromide) reagent (Sigma, St Louis, MO, USA) according to the manufacturer's instructions. Briefly, add reconstituted MTT in an amount equal to 10% of the culture medium volume. Return to incubator for 2 h. After that, medium was moved, and MTT formazan was resolved in 100 ul acidic isopropanol. Absorbance was measured at a wavelength of 570 nm. Experiments were performed in triplicate, and 3 different experiments were performed for each experimental condition. A cell proliferation curve was constructed by measuring cell growth for 4 consecutive days.

### Cell cycle assay

Cell cycle distribution was monitored by flow cytometry (FACSCalibur, Becton Dickinson, Franklin Lakes, NJ, USA) using propidium iodine (PI) staining (Becton Dickinson) according to the protocol of the manufacturer. Briefly, the H0-8910 PM cells which were pre-infected with shRNA-PDCD6 for 72 h were plated in 6-well plates. After cultured for 48 h, cells were collected, washed with PBS, and fixed with 2% paraformaldehyde at room temperature for 30 min. After paraformaldehyde was moved, cell pellets were resuspended by 70% ethanol and stored at -20°C for 2 h. Then spin cells out of fix, pellets were resuspend in PBS with 200 ug/ml RnaseA and incubated at 37°C for 1 h. Added PI to a final concentration of 50 ug/ml for 2 h before analyzing on the flow cytometer.

Software-based cell cycle analysis was performed using ModFit 2.0 (Verity, Topsham, ME).

### Hoechst stain for detecting apoptotic cells

Apoptotic cells were detected by fluorescence imaging using Hoechst 33258 stain (Sigma-Aldrich, St. Louis, MO, USA). Seventy-two hours after transfection, cells were washed twice with PBS, and then incubated with Hoechst reagent (10 mg/ml) for 30-45 min at 37°C in the dark. Morphological changes including a reduction in volume and nuclear chromatin condensation were observed under a fluorescence microscope (Nikon, Tokyo, Japan) and photographed at a magnification of 200×. Apoptotic cells were counted in at least three to four random fields and expressed as percentage of total cells.

### Transwell migration and invasion assay

For transwell migration assays, cells were plated in the top chamber with the non-coated membrane (24-well insert; pore size, 8 mm; BD Biosciences). For invasion assays, cells were plated in the top chamber with Matrigel-coated membrane (24-well insert; pore size, 8 mm; BD Biosciences). In both assays, a number of 2 × 10^5 ^HO-8910 PM cells treatment in 100 μL serum-free medium were pre-infected with shRNA-PDCD6 for 72 h and added to the upper chamber, while 500 μL medium with 10% FBS were added to the lower chamber. Transwells were incubated for 24 h at 37°C. Cells on the inside of the transwell inserts were removed with a cotton swab, and cells on the underside of the insert filter were fixed and stained. Photographs of four random fields were taken, and the cells were counted to calculate the average number of cells that had transmigrated.

### Patients and follow-up

Patient samples of ovarian cancer were consecutively collected in the Gynecologic Oncology Unit at University of Turin in Italy between October 1991 and February 2000 and all participants gave informed consent. Use of tissue samples was approved with an Ethical Review Committee of University. All ovarian cancer tissues were obtained at the time of cytoreductive surgery. In this study, we identified 212 patients with primary epithelial ovarian cancer who underwent cytoreductive surgery. The average patient age at surgery was 57.6 years (SD, 11.5; range, 26-82). Follow-up information was available for 203 patients who were followed from surgery to June 2001. The overall follow-up time ranged from 0.6 to 114 months, and the median was 31 months. The median disease progression-free survival was 20.6 months. Disease staging was classified according to the criteria of FIGO (the International Federation of Gynecologists and Obstetricians)[11]. Of the 211 patients with information on disease stage, 51 (24.2%) were diagnosed with stage I disease, 12 (5.7%) were stage II, 133 (63.0%) were stage III, and 15 (7.1%) were stage IV. Histological type determined following the World Health Organization (WHO) criteria [12] included serous, endometrioid, mucinous, clear cell, and other epithelial tumors. For data analysis, tumor histotypes were grouped into non-serous (59.9%, n = 127) and serous (40.1%, n = 85). Of the 211 patients with information on tumor grade, most patients (65.4%, n = 138) had grade 3 tumors (poorly differentiated); few had grade 1 or 2 (34.6%, n = 73). After surgery, most of the patients were treated with platinum-based chemotherapy following a standard protocol.

### Statistical analysis

The data from in vitro experiments were expressed as the mean ± SD for 3 independent experiments using three difference preparations. The difference between the means was assessed by Student's *t*-test. For patient study, *PDCD6 *mRNA expression was analyzed as a continuous variable and as a categorical variable after grouping by expression index (EI) distribution as low expression (EI < 0.1), middle expression (EI 0.1-1) and high expression (EI > 1). Associations between *PCDC6 *expression and clinical variables were analyzed using the Chi-square test. Survival analysis was performed for progression-free and overall survival using the Cox proportional hazards regression and Kaplan-Meier curves. All *p*-values were two-sided, and a probability of 0.05 or smaller was considered statistical significance. SPSS version 11.0 (SPSS Inc., Chicago, IL) was used for data analysis.

## Results

### Expression of PDCD6 in ovarian cancer cell lines with different metastatic potential

**Figure 1:**
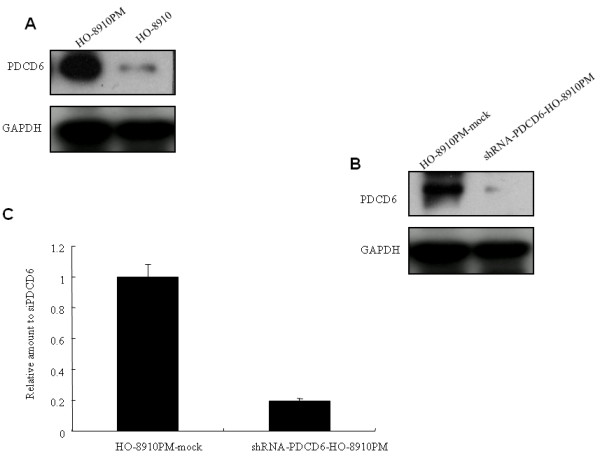
**A. Western blotting analysis showed protein expression of PDCD6 was higher in HO-8910 PM cells than that in HO-8910 cells**. GAPDH was used as a loading control. B. Western blotting analysis showed protein expression of PDCD6 in shRNA-PDCD6-HO-8910 PM was decreased compared with that in control HO-8910 PM-mock. GAPDH was used as a loading control. C. qRT-PCR results showed mRNA expression of *PDCD6 *in shRNA-PDCD6-HO-8910 PM was inhibited by 80% compared with that in HO-8910 PM-mock.

To confirm our previous proteomic results [6], we compared the expression of PDCD6 in H0-8910 and H0-8910 PM cells by western blot. The results showed that PDCD6 expression was higher in H0-8910 PM than that in H0-8910 (Figure 1A).

### Knockdown of *PDCD6 *in HO-8910 PM cells by lentiviral vectors-mediated transfection

In the present study, lentiviral vectors-mediated transfection was used to determine effects of *PDCD6 *expression silencing on metastatic ovarian cancer cell behavior. Seventy two hours after transfection, HO-8910 PM cells were observed using a fluorescent microscope. The transfection efficiency was evaluated by counting the green fluorescent cells, which was over 95% when the cells were infected with a multiplicity of infection (MOI) of 3. mRNA and protein levels of PDCD6 were detected by RT-PCR and Western blot at 72 h after transfection. The results showed pGCSIL-GFP-shRNA-PDCD6 lentiviral vector was effective in silencing PDCD6 expression and the inhibition rate of PDCD6 was about 80% (Figure 1B-C).

### Influence of HO-8910 PM cell proliferation, apoptosis and cell cycle by PDCD6 expression silencing in vitro

**Figure 2:**
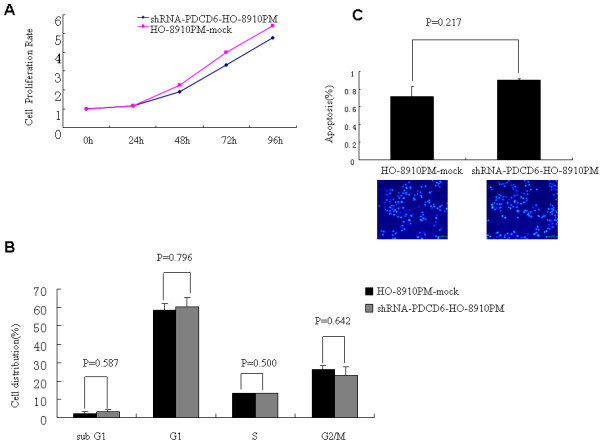
**A. Growth curve of HO-8910 PM cells infected with control or siRNA-PDCD6**. At each indicated time point, cell viability was determined and represented as the degree of absorbance 570 nm using the MTT. The mean ± SD absorbance (triplicate wells) for each time point is plotted as a function of the number of days after seeding. **B**. Cell cycle distribution in shRNA-PDCD6-HO-8910 PM and HO-8910 PM-mock cells. **C**. Apoptosis analysis in shRNA-PDCD6-HO-8910 PM and HO-8910 PM-mock cells by Hoechst 33258 staining. Normal cells showed normal nuclei blue, while apoptotic cells showed the cell nucleus was chunky, stainded densely and brightly.

As shown in Figure 2, PDCD6 knockdown had no obvious effect on cell proliferation and apoptosis although apoptotic cells were increased slightly and cell growth slowed down a little bit in HO-8910 PM cells infected with shRNA-PDCD6. No significant difference of cell cycle distribution was found.

### Inhibition of HO-8910 PM migration and invasion in vitro by siRNA-mediated PDCD6 knockdown

**Figure 3:**
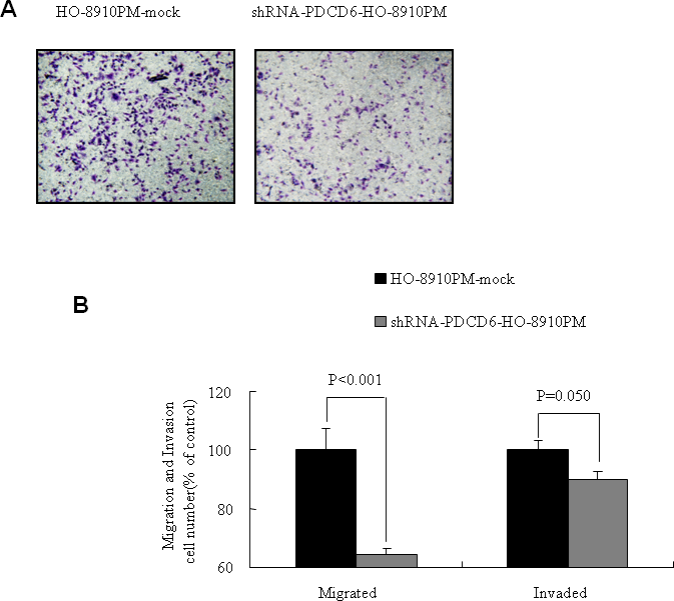
**A. Migrated cells in shRNA-PDCD6-HO-8910 PM and HO-8910 PM-mock cells**. Magnification in × 100. **B**. Quantitative analysis for migration and invasion assays of HO-8910 PM infected with shRNA-PDCD6 or control.

Migration and invasion assay in vitro were carried out to evaluate PDCD6 knockdown on HO-8910 PM cell motility. The result showed that the average number of migrated and invaded HO-8910 PM cells transfected with siRNA significantly decreased in comparison with the Mock group, *p *< 0.001 and *P *= 0.050 respectively (Figure 3).

### Correlation of *PDCD6 *mRNA expression with disease characteristics and patient survival

Spearman correlation analysis showed *PDCD6 *expression was positively correlate with residual tumor size (r = 0.16, *p *= 0.019) (Table 1). Compared the median expressions of *PDCD6 *by different clinical and pathologic characteristics, no significant difference was found, although *PDCD6 *expression was slight higher in advanced (stagesIII and IV) than that in early disease (stages I and II), 0.28 versus 0.16, *p *= 0.183, and in poorly differentiated tumors (grade3) than in well-differentiated tumors (grade 1 or 2), 0.29 versus 0.16, *p *= 0.275. Patients who had residual tumor after surgery had higher *PDCD6 *expression than patients who had no residual tumor, 0.31 versus 0.18, *p *= 0.261 (Table 2). Similar results were found when categorical variable was analyzed (Table 2), but significant difference of *PDCD6 *expression was showed between the patients with residual tumor and without residual tumor after surgery. Patients with residual tumor had significantly higher *PDCD6 *expression than patients who had no residual tumor (29.3% versus 16.7%, *P *= 0.050).

**Table 1:** Spearman correlation of PDCD6 mRNA expressions with clinicopathologic features of ovarian cancer

Variable	PDCD6 mRNA expression
	
	correlation coefficient (*P*)
Age (n = 209)	-0.03 (0.642)

Disease stage (n = 211)	0.07 (0.310)

Tumor grade (n = 211)	0.02 (0.748)

Tumor histology (n = 212)	0.00 (0.954)

Residual tumor size (n = 206)	**0.16 (0.019)^a^**

^a ^Bold values are statistically significant (*p *< 0.05)

**Table 2:** Association of PDCD6 mRNA expression with clinical and pathological characteristics of ovarian cancer

		*PDCD6 *Expression					
**Variable**	**n**	**Median (5th-95th)^a^**	***p-*value^b^**	**Low^c^**	**Middle^d^**	**High^e^**	***p-*value^f^**

**Stage**	**211**			**n (%)**	**n (%)**	**n (%)**	

I-II	63	0.16 (0-4.78)	0.183	25(39.7)	28(44.4)	10(15.9)	0.256

III-IV	148	0.28 (0-5.26)		52(35.1)	57(38.5)	85(26.4)	

Grade	211						

1~2	73	0.16 (0-4.41)	0.275	26(35.6)	33(45.2)	14(19.2)	0.477

3	138	0.29 (0-5.53)		51(37.0)	52(37.7)	35(25.4)	

Histology	212						

Non-Serous	127	0.20 (0-5.03)	0.321	47(37.0)	53(41.7)	27(21.3)	0.734

Serous	85	0.28 (0-5.41)		30(35.3)	33(38.8)	22(25.9)	

Residual Tumor	206						

No Residual	90	0.18 (0-3.69)	0.261	32(35.6)	43(47.8)	15(16.7)	***0.050 *^h^**

Residual	116	0.31 (0-7.20)		43(37.1)	39(33.6)	34(29.3)	

^a ^Expression levels at the 5th and 95th percentiles;^b ^Wilcoxon two-sample test with t approximation;^c ^Low: Expression Index (EI) < 0.1;

^d ^Middle: Expression Index (EI) 0.1-1;^e ^High: Expression Index (EI) > 1;^f ^*p-*value from Chi-square test;

^g ^*p-*value from Spearman correlation test;^h ^Bold values are statistically significant (*p *< 0.05)

**Figure 4:**
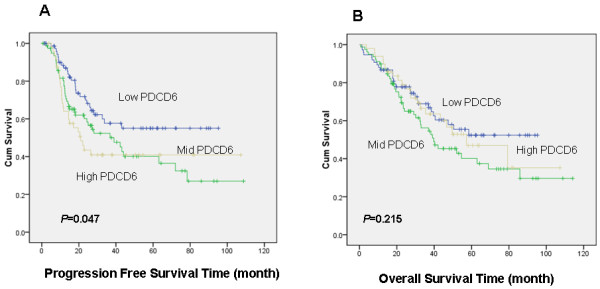
**Kaplan-Meier progression-free survival curves (A) and overall survival curves (B) according to *PDCD6 *levels: Among the patients, 77 patients had low *PDCD6 *expression, 86 patients had medium *PDCD6 *expression, and 49 patients had high *PDCD6 *expression**.

*PDCD6 *mRNA expression was significantly associated with progression free survival (Table 3). Patients with medium or high levels of *PDCD6 *mRNA were at higher risk for disease progression, compared to those with low levels (HR, 1.29; *P *= 0.024 for mid levels; and HR, 1.57; *P *= 0.045 for high levels) after adjusting for age, disease stage, tumor grade, histologic type and residual tumor size. Kaplan-Meier survival analysis demonstrates similar results. Patients who had medium or high *PDCD6 *mRNA had worse progression free survival (Figure 4). No significant association was found between *PDCD6 *mRNA expression and overall survival although patients with medium or high levels of PDCD6 mRNA were at higher risk for death compared to those with low level.

**Table 3:** Associations of PDCD6 mRNA expression and patient survival^a^

	Crude HR	95% CI^b^	*p-*value	Adjust HR	95% CI	*p-*value
Disease-Free Survival^c^						

Low PDCD6	1			1		

Mid PDCD6	1.65	1.01-2.70	***0.046***	1.29	1.08-2.91	***0.024***

High PDCD6	1.87	1.09-3.21	***0.023***	1.57	1.09-2.58	***0.045***

Overall Survival^d^						

Low PDCD6	1			1		

Mid PDCD6	1.51	0.93-2.43	0.094	1.88	0.95-3.08	0.092

High PDCD6	1.13	0.64-1.99	0.664	1.07	0.61-1.91	0.801

^a ^Associations determined by Cox proportional hazards regression and adjusted for age, stage, grade, histotype and residual tumor size. Significance level of *p *= 0.05

^b ^95% Wald Confidence Limits

^c ^Hazard Ratio (HR) for relapse with respect to low PDCD6 expression

^d ^Hazard Ratio (HR) for death with respect to low PDCD6 expression

## Discussion

In our proteomic analysis of ovarian cancer cell lines, we detected 21 significantly different spots (two-fold increase or decrease) through 2-D gel electrophoresis, of which 17 candidate proteins were successfully identified and characterized. These proteins were mainly involved in the regulation of cell growth, proliferation, motility, apoptosis and tumor immunity [6]. To further study the function of those differentially expression proteins in ovarian cancer, in this study, we selected PDCD6, which had the highest protein score and 100% protein confidence interval [6]. As expected, PDCD6 overexpression in HO-8910 PM as compared to HO-8910 was confirmed by western blot analysis.

PDCD6, discovered by cDNA library screening for apoptosis-related genes [13], is a calcium binding protein which belongs to the penta-EF-Hand family that has the Ca2 + -binding helix-loop-helix structures [14]. PDCD6 functions as a Ca2+ sensor by changing its conformation [15]. This conformational change enables PDCD6 to interact with various intracellular proteins containing Pro-rich regions in a Ca2 + -dependent manner, such as Alix (ALG-2-interacting protein X)[16], annexins VII, XI [17], Sec31A (SEC31 homolog A)[18], and TSG101 (tumor susceptibility gene 101)[16]. Original cloning report of PDCD6 cDNA in a screen for genes involved in apoptosis [13] and early studies indicated PDCD6 participates in T cell receptor-induced programmed cell death in a Ca2 + -dependent manner as a pro-apoptotic factor [19]. Recent study showed Alix and ALG-2 involved in tumor necrosis factor receptor 1-induced cell death by interacting with pro-caspase-8 [20]. However, apoptosis was not blocked in PDCD6 deficient mice, suggesting that PDCD6 is functionally redundant [21]. Furthermore, PDCD6 was found to be up-regulated in a variety of human tumors compared to normal tissues of the breast, liver, lung, and colon, especially in metastatic tissues, which suggests that in addition to its known pro-apoptotic function PDCD6 may play a role in cell survival [22-24]. Additionally, in vitro experiments indicated that PDCD6 had an anti-apoptotic action in HeLa cells via facilitating proliferating cells passing through the G2/M cell cycle checkpoints [25].

Different from the previous study, PDCD6 knockdown had no obvious effect on cell proliferation and apoptosis although apoptotic cells were increased slightly and cell growth slowed down a little bit in HO-8910 PM cells infected with shRNA-PDCD6, but did result in decrease in cell motility and invasiveness. These results indicated that over expression of PDCD6 promote both migration and invasion in ovarian cancer cells.

Significantly, our patient study supported the results in vitro. *PDCD6 *mRNA expression was significantly correlated with residual tumor size. Patients with residual tumor had higher *PDCD6 *expression than patients with no residual tumor. Furthermore, *PDCD6 *expression was associated with patient survival; patients with high *PDCD6 *mRNA had shorter disease progression-free survival than those with middle or low expression. Our findings suggest that *PDCD6 *mRNA expression is an independent predictor of ovarian cancer progression free survival.

In the study, we found that high PDCD6 expression was associated with disease progression, but not death. There are a number of possible reasons that may explain this discrepancy. First, PDCD6 may promote disease progression or reduce the effect of adjuvant treatment by stimulating tumor cell migration or invasion, but these effects do not lead to the death of all cases. Some patients may die of other reasons. Second, the study was relatively small and the follow-up time was short. We do not have enough power to find a significant association with overall survival. Third, our study findings are still preliminary. Independent large studies are needed to confirm these results.

These findings may be explained by our observations in vitro and vivo which are generally in agreement with the findings of other studies. One study of lung cancer demonstrated that PDCD6 overexpression was indicative of unfavorable prognosis for patients with early stage non-small cell lung cancer or lung adenocarcinoma; the protein may serve as a potential molecular marker for aggressive lung cancer [23]. However, no association was found between PDCD6 protein expression measured by immunohistochemical staining and survival of lung, breast or colon cancer patients [26]. And recent study reported that purified recombinant human PDCD6 inhibits vascular endothelial growth factor (VEGF)-induced proliferation, invasion, and capillary-like structure tube formation in vitro through PI3K/mTOR/p70S6K pathway by interacting of VEGFR-2 [27]. Those inconsistent findings in the literature may be due to the observations made on different cancer cell lines or disease stages. In early stage tumors or tumors other than ovarian cancer, PDCD6 may have a pro-apoptotic effect on the tumors, whereas in our case when the tumor is ovarian cancer or tumors progress to advanced stages (since most of ovarian cancers are grade 3 tumors), the PDCD6 function changes to an opposite direction either due to different regulation or downstream targets, which is anti-apoptotic or pro-migration that leads to shortened progression-free survival.

In the future, we will continue this work from clinic to confirm its prognostic utility as well as from lab experiment to elucidate its molecular mechanism, finding or assembling another clinical study to measure PDCD6 expression in ovarian tumors and to analyze its association with disease progression and overall survival, performing chip assay to identify upstream modulators of PDCD6 as well as its downstream targets or pathways being involved in the PDCD6's effect on tumor progression. With a comprehensive understanding of PDCD6 regulation and function, we may be able to tell what confounding biology is involved in the relationship between PDCD6 expression and ovarian cancer progression.

## Conclusions

In summary, PDCD6 was identified and characterized as differentially expressed proteins between ovarian cancer cells with low and high metastatic property by our previous comparative proteomic study. It was further confirmed to be overexpressed in HO-8910 PM as compared to HO-8910 by western blot. PDCD6 knockdown significantly inhibited migration and invasion of ovarian cancer cells in vitro, supporting the notion that PDCD6 played an important role in ovarian cancer progression. *PDCD6 *mRNA was detectable in most of ovarian tumor samples, and the expression was correlated with residual tumor size. High *PDCD6 *expression was found to be associated with unfavorable prognosis of the disease. It may serve as an independent marker for ovarian cancer prognosis. Further studies are needed to more completely elucidate the molecular mechanisms of PDCD6 in ovarian cancer progression.

## Competing interests

The authors declare that they have no competing interests.

## Authors' contributions

XHY, FJG and GLH carried out the cell assay; YLS and GY carried out western blot and RT-PCR assay; SD, KD and YH carried out the case collection; and SD and XHY analyzed the results.XSH and QM conceived the study, participated in the design, and coordinated and helped draft the manuscript. All authors read and approved the final manuscript.
